# PD-1 Cellular Nanovesicles Carrying Gemcitabine to Inhibit the Proliferation of Triple Negative Breast Cancer Cell

**DOI:** 10.3390/pharmaceutics14061263

**Published:** 2022-06-14

**Authors:** Hualian Zha, Zhanxue Xu, Xichao Xu, Xingyu Lu, Peilin Shi, Youmei Xiao, Hsiang-I Tsai, Dandan Su, Fang Cheng, Xiaoli Cheng, Hongbo Chen

**Affiliations:** 1 School of Pharmaceutical Sciences (Shenzhen), Shenzhen Campus of Sun Yat-sen University, Shenzhen 518107, China; zhahlian@mail2.sysu.edu.cn (H.Z.); 15602493072@163.com (Z.X.); luxy86@mail2.sysu.edu.cn (X.L.); shiplin@mail2.sysu.edu.cn (P.S.); xiaoym9@mail2.sysu.edu.cn (Y.X.); tsaihsangi88@163.com (H.-I.T.); sudd6@mail2.sysu.edu.cn (D.S.); 2 Department of Pharmacy, Guangzhou Institute of Respiratory Health, First Affiliated Hospital of Guangzhou Medical University, Guangzhou 510120, China; 3 Endoscopy Center and Gastroenterology Department, Key Laboratory for Precision Diagnosis and Treatment of Pediatric Digestive System Diseases, Shenzhen Children’s Hospital, Shenzhen 518036, China; xichaoxu0@163.com; 4 Department of Pharmacy, Shenzhen Bao’an Maternal and Child Health Hospital, Shenzhen 518133, China

**Keywords:** PD-1, nanovesicles, immunotherapy, gemcitabine, breast cancer

## Abstract

PD-1 inhibitor Keytruda combined with chemotherapy for Triple-negative breast cancer (TNBC) has been approved for FDA, successfully representing the combination therapy of immunotherapy and chemotherapy for the first time in 2020. However, PD-L1 inhibitor Tecentriq combined with albumin paclitaxel using the similar strategy failed to achieve the expected effect. Therefore, it is still necessary to explore new effective immunotherapy and chemotherapy-based combined strategies. We developed a cell membrane-derived programmed death-ligand 1(PD-1) nanovesicle to encapsulate low-dose gemcitabine (PD-1&GEM NVs) to study the effect on breast cancer in vitro and in vivo. We found that engineered PD-1&GEM NVs could synergistically inhibit the proliferation of triple-negative breast cancer, which interacted with PD-L1 in triple-negative breast cancer to disrupt the PD-L1/PD-1 immune inhibitory axis and promoted cancer cell apoptosis. Moreover, PD-1&GEM NVs had better tumor targeting ability for PD-L1 highly-expressed TNBC cells, contributing to increasing the drug effectiveness and reducing toxicity. Importantly, gemcitabine-encapsulated PD-1 NVs exerted stronger effects on promoting apoptosis of tumor cells, increasing infiltrated CD8^+^ T cell activation, delaying the tumor growth and prolonging the survival of tumor-bearing mice than PD-1 NVs or gemcitabine alone. Thus, our study highlighted the power of combined low-dose gemcitabine and PD-1 in the nanovesicles as treatment to treat triple-negative breast cancer.

## 1. Introduction

By 2020, the number of new cases and deaths of female breast cancer has surpassed that of lung cancer and it has become the most common cancer [1]. Triple-negative breast cancer (TNBC) is a highly heterogeneous disease that constitutes almost 20% of breast cancer cases, which is associated with poor overall survival and a high probability of distant recurrence and death [2,3]. In the past 10 years, surgical treatment is still the first-choice strategy for early breast cancer patients.

In addition to resection, chemotherapy is the cornerstone of systemic therapy for TNBC. Anthracycline, taxanes, and platinum drugs are still the most basic chemotherapy regimens. Meanwhile, the role of Gemcitabine has also been highlighted [4]. Gemcitabine, a cell cycle-specific antimetabolite, is characterized by wide antitumor spectrum and few adverse reactions. In recent years, several large-scale phase III studies have shown that single or combined chemotherapy drugs are effective for advanced breast cancer. Many studies have shown that gemcitabine is effective in the treatment of advanced triple-negative breast cancer patients. The clinical remission rate of single drug is 15–20% and if combined anthracycline and taxanes, the overall remission rate can be increased to 20–79% [5], which makes it the first-choice chemotherapy drug as combination drug recommended for advanced breast cancer [6]. However, the increased toxic reaction and resistance of gemcitabine following the continuous implementation of the combined treatment have become obstacles that limit the in-depth application of gemcitabine in breast cancer. Therefore, exploring new effective combination therapy and reducing side effects through targeted administration are still of great clinical significance to expand the application of Gemcitabine.

Programmed death 1 (PD-1) is an immunosuppressive member on the surface of T cells and plays a vital role in regulating the immune system. PD-1 maintains immune homeostasis upon binding to its ligands, PD-L1 or PD-L2 [7]. However, tumors utilize this immune protective mechanism for their immune survival. In the tumor microenvironment, tumor cells and tumor-associated APCs highly express PD-L1, which combines with PD-1 of T cells to induce T cell exhaust, thereby inhibiting the anti-tumor function of CD8^+^ T cells [8]. Therefore, destroying the interaction between PD-L1/PD-1 shows great potential in enhancing the lethality of the immune system to cancer cells [9,10]. In recent years, PD-1/PD-L1 blocking antibody drugs have made a breakthrough in tumor immunotherapy [11]. At present, several antibody drugs have been approved for clinical application in a variety of fields such as melanoma, non-small cell lung cancer, Hodgkin’s lymphoma, and have achieved good results [12]. Excitedly, the FDA recently approved PD-1 inhibitor Pembrolizumab (Keytruda) combined chemotherapy (Albumin paclitaxel/paclitaxel with or without carboplatin) as a new adjuvant therapy for the unresectable or metastatic triple-negative breast cancer (TNBC) with PD-L1 expression in ≥10% tumor cells. In the final analysis of phase III keynote-355 trial, pembrolizumab plus chemotherapy significantly improved the progression-free survival (PFS) compared with chemotherapy alone, which suggests that chemotherapy combined with immunotherapy may be a promising treatment option for TNBC [13,14]. However, in contrast, because the clinical trials of PD-L1 monoclonal antibody inhibitor Atezolizumab (Tecentriq) are not enough to prove that the drug combined with other chemotherapeutic drugs is better than monotherapy [3], the European Drug Administration (EMA) withdrew Roche’s application of the combination therapy of Atezolizumab with albumin paclitaxel or anthracyclines in the treatment of early or advanced TNBC [15]. These two contradictory results indicated that it is still very necessary to explore new combination therapies of different immune drugs and chemotherapeutic drugs.

Extracellular vesicles are important carriers for transferring information and substances between cells [16], and they play a significant role in tumor immune escape [17] and tumor metastasis [18]. Studies have shown that nucleic acid therapeutics can be transported to breast cancer tumors expressing EGFR (epidermal growth factor receptor) through extracellular vesicles [19] and may have anti-metastatic effects [20]. Importantly, cell membrane-derived nanovesicles (NVs) with serial extrusion have similar characteristics to the exosomes naturally secreted by cells and also inherit the characteristics of innate cells. NVs have several superiorities in large-scale preparation and expressing complex active proteins as a compound carrier to release the drug at target sites by the ways of co-incubation and electroporation [21,22].

Thus, we constructed an extracellular vesicle-like cell membrane nanovesicle (NVs) to co-deliver PD-1 and gemcitabine. NVs have the superior characteristics of excellent biocompatibility, large-scale preparation, and strong drug delivery capabilities [23,24]. They can not only carry targeted proteins or ligands stably through genetic modification strategies on the membrane of NVs, but also carry various traditional drugs inside the NVs, which can increase the ability of NVs to target tumor and reduce the systemic side effects of drugs. The cell membrane vesicles have the promising potential to become an ideal nano-scale delivery system.

In this study, we established PD-1-expressing NVs carrying low-dose gemcitabine to suppress the immune escape pathway, thus inhibiting tumor growth. The combination of PD-1 NVs and gemcitabine significantly promoted the peripheral blood mononuclear cell (PBMC) activation, inhibited the proliferation of triple-negative breast cancer, induced the cancer cell apoptosis, increasing the infiltrated CD8^+^ T cells, delaying the tumor growth, and prolonging the survival of tumor-bearing mice. Taken together, these data supported the idea that the power of combined low-dose gemcitabine and PD-1 in the nanovesicles presented a prospective strategy to treat triple-negative breast cancer.

## 2. Materials and Methods

### 2.1. Chemicals and Regents

Puromycin was supplied by Sigma-Aldrich (Merck, St. Louis, MO, USA). Na^+^/K^+^ ATPase antibodies were obtained from Santa Cruz (Santa Cruz Biotechnology, Santa Cruz, CA, USA). Primary antibodies such as PD-1, GFP, and β-actin for western blotting were purchased from Cell Signaling Technology (Cell Signaling Technology, Danvers, MA, USA). Wheat germ agglutinin (WGA) Alexa Fluor 594 and 350 dyes were purchased from Thermo Scientific (ThermoFisher, Waltham, MA, USA). Staining antibodies including CD3, CD4, CD8, and CD25 for fluorescence activated cell sorting (FACS) analysis were purchased from Biolegend Inc. (BioLegend, San Diego, CA, USA)**,** Ficoll Paque Plus was purchased from GE Healthcare (GE Healthcare, Chicago, IL, USA).

### 2.2. Plasmids

Human PD-1 lentivirus open reading fragment (ORF) cDNA expression plasmid with green fluorescent protein (C-GFP Spark tag) and mouse PD-1 lentivirus ORF cDNA expression plasmid (C-GFP Spark tag) were supplied by Sino Biological Inc (Sino Biological, Beijing, China).

### 2.3. Cell Lines

MDA-MB-231 cells (SCSP-5043), HEK293T cells (SCSP-502), MCF-7 cells (SCSP-531), 3T3L1 cells (SCSP-5038), and 4T1 cells (TCM32) were purchased from Chinese Academy of Sciences Cell Bank (Shanghai, China). MDA-MB-231 cells, MCF-7 cells and 4T1 cells, HEK293T cells and 3T3L1 cells were separately cultured in Leibovitz (L-15) (HyClone, Logan, UT, USA), minimum eagle’s medium (MEM) (HyClone, Logan, UT, USA), Roswell Park Memorial Institute (RPMI-1640) (HyClone, Logan, UT, USA) and Dulbecco’s modified eagle medium (DMEM) (HyClone, Logan, UT, USA) with 100 U/mL penicillin, 100 μg/mL streptomycin and 10% fetal bovine serum (FBS) (ExCell Bio, Canelones, Uruguay), at 37 °C with 5% CO_2_.

In order to obtain HEK293T/3T3L1 cell lines that stably overexpress PD-1, HEK293T cells (1.5 × 10^7^) were firstly used to produce viral particles (10^7^ transducing units (TU)/mL) in the lentivirus packaging solution environment (phosphate buffer saline (HBS) solution 950 µL, packaging plasmid 10 µg, human/mouse PD1 gene lentiviral ORF cDNA expression plasmid 10 µg, 2.5 M CaCl_2_ solution 50 µL). HEK293T/3T3L1 cells were then infected with collected viral particles at 12 h, 24 h, and 48 h post-transfection and co-incubated for 24 h at 37 °C. Under selection with puromycin, HEK293T/3T3-L1 cell lines with a high degree of overexpressing PD-1-GFP on the cell membrane was finally obtained.

### 2.4. Western Blotting

Cell samples and cell vesicles samples were lysed in radio immunoprecipitation assay lysis buffer (RIPA) solution, and then subjected to 10% SDS-PAGE for electrophoresis at 120 v and western transfer at 330 mA, 90 min. Primary antibodies of Na^+^/K^+^ ATPase, PD-1, GFP and β-actin were co-incubated overnight at 4 °C. After incubation with horseradish peroxidase (HRP) bounded anti-mouse or anti-rabbit secondary antibodies (Protein Tech, Wuhan, China), target proteins were detected by enhanced chemiluminescence (ECL) kit (Protein Tech, Wuhan, China).

### 2.5. Cell Membrane Vesicle Preparation

The collected HEK293T/3T3-L1 cells (3 × 10^7^) were fully cracked in 2 mL homogenization medium buffer (1 mM EDTA, 0.25 M sucrose, 20 mM Hepes-NaOH, pH 7.4, and protease inhibitor cocktail), then thoroughly ground with a grinder, and centrifuged at different speeds of 5000 rpm (4 °C, 10 min) and 13,500 rpm (4 °C, 10 min), respectively. After filtration through 0.45 μm and 0.22 μm filters, the obtained vesicles were stored at −80 °C.

### 2.6. Size Distribution, Zeta-Potential Analysis, and Morphology

HEK293T/3T3-L1 cell membrane (3 × 10^7^ cells) were dissolved with 0.5 mL PBS and filtered by 0.1 μm filter. The particle size distribution and zeta potential range of the samples were determined by a NanoBrook 90Plus PALS instrument (Brookhaven, NY, USA). The morphology of nanovesicles stained with uranium acetate on copper mesh were scanned by transmission electron microscopy (TEM) (JEOL, Tokyo, Japan).

### 2.7. Nanovesicle Cell Binding Assay

MDA-MB-231 cells were co-incubated with GFP-PD-1 NVs (25 mg/mL) for 2 h at 37 °C, and then stained with WGA Alexa594 for 10 min. After washing with phosphate buffer saline, images were captured using LSM880 confocal microscope (Zeiss, Jena, Germany) emitted by corresponding lasers 594.

### 2.8. Isolation of Peripheral Blood Mononuclear Cell (PBMC) from Human Peripheral Blood

Blood from healthy donors was collected into EDTA empty bottles, and then Ficoll Paque Plus (GE Healthcare, Chicago, IL, USA) separation solution was added. The blood was centrifuged at 1000 rpm for 30 min and PBMCs were obtained. PBMCs were washed twice with RPMI 1640, and lysed on ice for 2 min with 1 mL erythrocyte lysates. Finally, RPMI 1640 was added to neutralize and resuspend PBMCs.

### 2.9. Production of GEM&PD-1 NVs

The PD-1 NVs (0.5 mg protein weight) and 1 mg GEM were mixed gently in 1 mL electroporation liquid (1.15 mM potassium phosphate, pH 7.2, 25 mM potassium chloride), and electroporation was carried out in 0.4 cm electroporation cuvette by using bio-RAD electroporation instruments (Bio-Rad, Hercules, CA, USA). Parameters: 300 V, 150 µF, 3 s. The sample was then left on ice for 30 min. After washing with cold PBS solution and centrifuging at 12,000 rpm, GFP-PD-1 NVs carrying GEM (GEM&PD-1 NVs) were obtained.

### 2.10. Detection of Encapsulation Ratio

To obtain the GEM standard curve, standard solutions of different concentrations (50 µg/mL, 40 µg/mL, 30 µg/mL, 20 µg/mL, and 10 µg/mL of 2 mL standard solutions) were prepared and the absorbance values were measured at 268 nm by ultraviolet spectrophotometer. After measuring the absorbance of GEM in the sample, the concentration was calculated by substituting the GEM calibration curve.

Encapsulation Ratio% E.R. = MG/MS × 100% (MG is the mass of GEM in the supernatant and MS is the total mass of GEM in the sample).

### 2.11. Carboxyfluorescein Diacetate Succinimidyl Easter (CFSE) Staining

PBMCs were spread in 24-well plates with CD3 antibody, and then CFSE dye (5 μM) was added. The PBMCs were co-incubated at 37 °C in 5% CO_2_ for 20 min under dark conditions, and then RPMI-1640 was added to stop response. After centrifugation (1000 rpm, 5 min) and repeated washing with RPMI-1640 medium, the stained cells were placed on 48-well plates with plate-bound anti-CD3 antibody (5 μg/mL) and allowed to grow for 2 days with the addition of the activating factor IL-2. MDA-MB-231 cells were co-incubated with PBMCs and vesicles of different groups (50 µg/mL). Then, apoptosis of MDA-MB-231 cells was detected by flow cytometry (Cytoflex, Beckman, Brea, CA, USA).

### 2.12. Biodistribution

NVs labelled by sulfo-cyanine5.5 amine (cy5.5) (excitation/emission peaks, 680 nm/710 nm) (Lumiprobe Corporation, Hallandale Beach, FL, USA) were injected into BALB/c mice through the tail vein. After 4 h, the distribution of NVs in the main organs of mice was observed by NightOWL (LB983) imaging system (Berthold, Pforzheim, Germany).

### 2.13. Breast Cancer Mouse Models

The use of laboratory animals and all experiments was reviewed and approved by the Animal Ethics Committee of Sun Yat-sen University, China, approval No. SYSU-IACUC-2021-000046.

Specific pathogen free (SPF) female BALB/C mice were purchased and adaptively fed at sterile room temperature for one week up to eight weeks for mouse models of breast cancer. Mice were firstly anesthetized by intraperitoneal injection of 1% pentobarbital, and the hair around the fourth pair of breasts were removed with shaving cream, and then disinfected with 75% alcohol. The prepared suspension of mouse breast cancer 4T1 cells (3 × 10^6^) was injected to the mammary fat pad. Seven days later, the mouse models of breast cancer were established successfully. All 25 tumor-bearing mice were randomly divided into 5 groups and received different treatments: Control group (normal saline, *n* = 5), Free nanovesicles group (Free NVs, 25 mg/kg, *n* = 5), PD-1 nanovesicles group (PD-1 NVs, 25 mg/kg, *n* = 5), single drug group (GEM, 10 mg/kg, *n* = 5), PD-1 nanovesicles and GEM group (PD-1&GEM NVs, 10 + 25 mg/kg, *n* = 5). In the first three days, mice were treated according to the above scheme every day. After three days, mice were treated every other day for continuous 10 days and sacrificed on the 10th day (on the 18th day from tumor inoculation). Tumor, spleens, and lymph nodes samples were collected and analyzed on the 18th day.

### 2.14. Haematoxylin and Eosin Staining

Paraffin sections of tumor and spleen tissue samples were dewaxed, then placed in graded concentrations of alcohol (100%, 95%, 90%, 80%, 70%) for hydration. Tissue sections were stained with H&E, and inflammatory cell infiltration was observed by microscopy (Nikon, Tokyo, Japan) with 100× magnification.

### 2.15. Cell Isolation from Spleen, Tumor, and Lymph Node and Flow Cytometry Assay

Spleens, tumors, and lymph nodes cells (10^6^) that were obtained after mouse dissection were infiltrated in PBS solution and the tissues were ground, washed in PBS solution, and passed through a 70 μm filter. All above operations were performed on ice to keep the cell activity. After 1000 rpm centrifugation for 5 min, the samples were co-incubated with 100 μL of the staining buffer and target antibodies of 1 μL, such as anti-CD3-FITC, anti-CD4-APC, and Anti-CD8-PB450, and shielded from light for 15 min at room temperature. After centrifugation and washing with PBS, samples should be performed by MoFlo XDP flow cytometer (Beckman Coulter, Miami, FL, USA).

### 2.16. Annexin V/Propidium Iodide-Based Cell Apoptosis Assay

The cell samples treated under different conditions were collected, centrifuged, and re-suspended with 100 μL 1 × Annexin V Binding buffer. Then, 5 μL Annexin V-FITC and 5 μL PI were added according to the Apoptosis Detection Kit (BD Pharmingen, San Diego, CA, USA), and gently mixed and incubated for 15 min at room temperature, away from light. Samples should be detected within 1 h using the MoFlo XDP flow cytometer (Beckman Coulter, Miami, FL, USA).

### 2.17. Cell Viability and Colony Formation Assays

For the cell viability assay, MDA-MB-231 cells were seeded into 96-well plates (5 × 10^4^) and incubated at 37 °C, 5% CO_2_ overnight. The IC50 analysis of GEM on MDA-MB-231 cells were treated with different doses of GEM for 48 h. The cell viability was assessed using CCK-8 according to the instructions. Experiments were repeated at least three times. For colony formation assay, MDA-MB-231 cells (1 × 10^3^/well) were seeded in 6-well plates and cultured in L-15 medium with various doses of GEM for 2 weeks. Then they were fixed by 4% paraformaldehyde (PFA) and stained with crystal violet for cell colony number count.

### 2.18. Quantitative Real-Time PCR

Total RNA from cells were isolated by 1 mL TRIZOL reagent (TaKaRa, Tokyo, Japan). Cells after washing with PBS were lysed in Trizol. Then, 200 μL trichloromethane, 550 μL isopropyl alcohol, and 1 mL 75% ethanol was added in turn, and 12,000 rpm centrifuge is required after each addition. Next, the 1 μg RNA was reversely transcribed into cDNA by using 4 μL HiScript III RT SuperMix for qPCR (+1 μL gDNA wiper) (Vazyme, Nanjing, China). Finally, RT-qPCR experiments were performed with 3-step amplification program (95 °C 10s, 60 °C 10s, 72 °C 10s) by using 2x SYBR Green qPCR Mix (TransGen Biotech, Beijing, China) with LightCycler^®^ 96 (Roche, Basel, Switzerland). The quantitative calculation analysis of relative gene expression could be determined by 2^−ΔΔCt^ method. Experiments were performed in triplicate.

### 2.19. Statistical Analysis

Data analysis was performed using GraphPad Prism version 7.0 software (GraphPad Software Inc., La Jolla, CA, USA) and Origin Pro 2021 software. One-way analysis of variance (ANOVA, R.A.Fisher) and Turkey’s test were performed. All data were presented as mean ± standard error (SEM). Statistical significance is indicated (* *p* < 0.05, ** *p* < 0.01, *** *p* < 0.001).

## 3. Results

### 3.1. Construction of Stably Overexpressing PD-1 Cell Lines and Biological Behaviors of PD-1 NVs In Vitro

**Figure 1:**
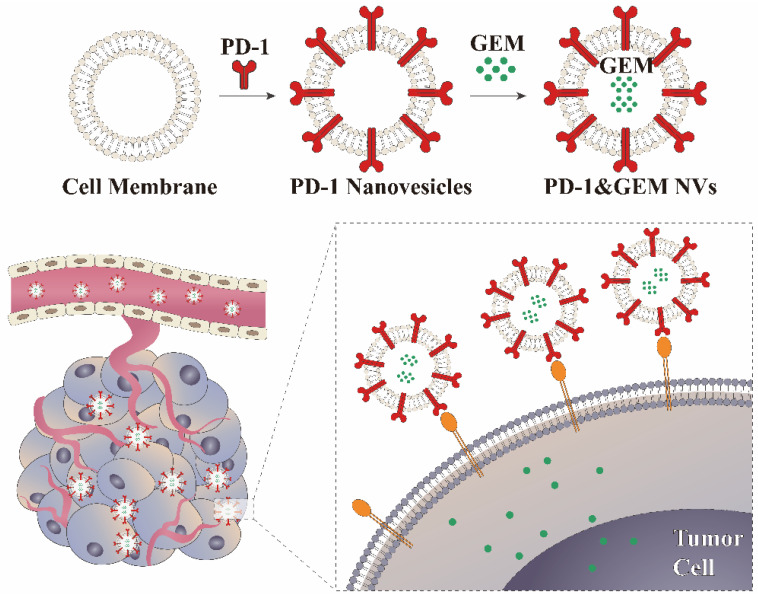
Schematic illustration of engineering PD-1 NVs carrying GEM for the treatment of triple-negative breast cancer.

As shown in Figure 1, we designed membrane-derived nanovesicles to carry PD-1 protein. PD-1 can bind to PD-L1 on the surface of breast cancer cells. Therefore, PD-1 NVs could block PD-1/PD-L1 immunosuppressive signaling pathways. To inhibit the proliferation of breast cancer cells, we used PD-1 NVs to encapsulate low-dose GEM drugs (PD-1&GEM NVs).

**Figure 2:**
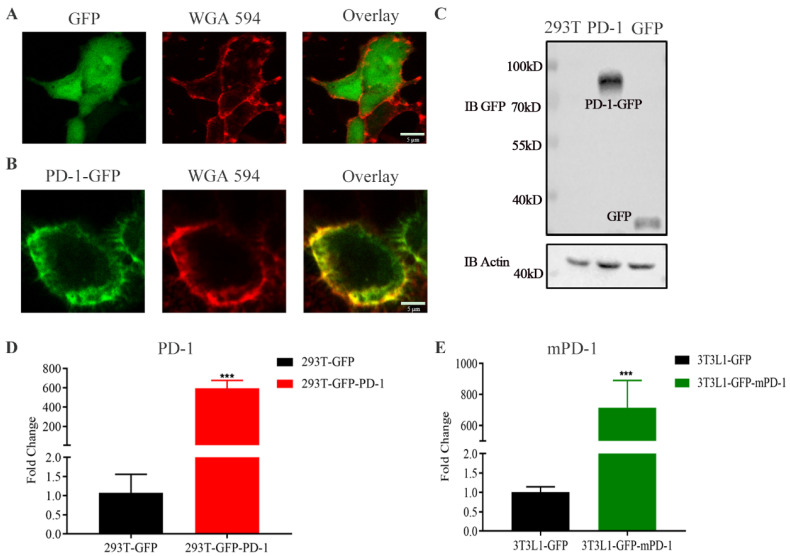
Schematic illustration and characterization of HEK293T/3T3L1 stably overexpressing PD-1. (**A**,**B**) Confocal images indicated the establishment of HEK293T cell line stably expressing GFP and human PD-1. WGA Alexa-Fluor 594 dye was used to label cell membrane. Scale bar: 5 µm. (**C**) Western blot assay verified the expression of PD-1 receptors on the whole cell lysate of the overexpressing PD-1 cell line. β-actin was used as a loading control. (**D**,**E**) RT-qPCR assay exhibited the expression of PD-1 on the whole cell lysate of the stable cell line including human cell (HEK293T) and mouse cell (3T3L1). Data were expressed as mean ± SEM, *n* = 3. *** *p* < 0.001.

To obtain PD-1 NVs, we firstly established PD-1-GFP stably overexpressing HEK293T cells using the lentiviral packaging system. HEK293 T cells were easy to proliferate with high transfection [21]. Thus, we constructed HEK293T cells derived PD-L1 NVs in vitro. Confocal microscopy showed the obvious cell membrane localization of PD-1-GFP (Figure 2B), while GFP as the control was mainly localized in the cytoplasm (Figure 2A). Furthermore, PD-1-GFP overexpression was confirmed in HEK293T stably cell lines by western blotting (Figure 2C). Compared with GFP control, the expression of PD-1-GFP mRNA was more than 600 times higher through the real-time quantitative PCR experiment (Figure 2D) (Table 1). At the same time, in order to meet the needs of subsequent treatment of transplanted tumors in mice to avoid cross-species reaction, we also constructed a mouse 3T3L1 cell line with high expression of PD-1-GFP, and confirmed its high expression by qPCR (Figure 2E) (Table 1).

**Figure 3:**
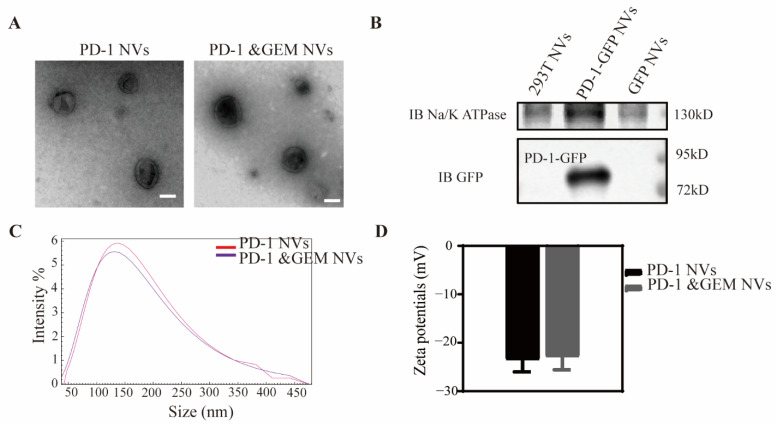
The preparation and characterization of PD-1 NVs and PD-1&GEM NVs. (**A**) The TEM image showed the shape and size of PD-1 NVs and PD-1&GEM NVs. Scale bar: 100 nm. (**B**) Western blot assay exhibited the expression of PD-1 receptors on the NVs of the stable cell line. Na, K-ATPase was used as a loading control. (**C**,**D**) The size distribution and zeta potentials of PD-1 NVs and PD-1&GEM NVs measured by dynamic light scattering (DLS) analysis. Data were expressed as mean ± SEM, *n* = 3.

To prepare HEK293T derived PD-1-GFP NVs and PD-1&GEM NVs, extruded nanovesicles were collected by cell lysis, differential centrifugation, and a serial extrusion as described in the Material and Method section. The cell vesicles were estimated by transmission electron microscope (TEM). The PD-1 NVs and PD-1&GEM NVs were found to be membrane-bound closed shape and about 100 nm (Figure 3A). In order to proof the expression of PD-1 in vesicles, we detected the PD-1 and Na^+^/K^+^ ATPase expression via western blotting. Results have shown that the PD-1 was expressed in cell vesicles (Figure 3B). Furthermore, dynamic light scattering (DLS) analysis also showed that PD-1-GFP NVs and PD-1&GEM NVs had a similar diameter with around 110 nm (Figure 3C), and the zeta potentials of PD-1-GFP NVs and PD-1&GEM NVs was around −22 mv (Figure 3D), indicating that the PD-1-GFP NVs and PD-1&GEM NVs were a stable membrane structure. Therefore, all data showed that we have successfully prepared the PD-1-overexpressing cell membrane-based nanovesicle and the final product of PD-1&GEM NVs.

### 3.2. PD-1-GFP NVs Specifically Bound to PD-L1 on the Surface of MDA-MB-231 Triple-Negative Breast Cancer Cells

**Figure 4:**
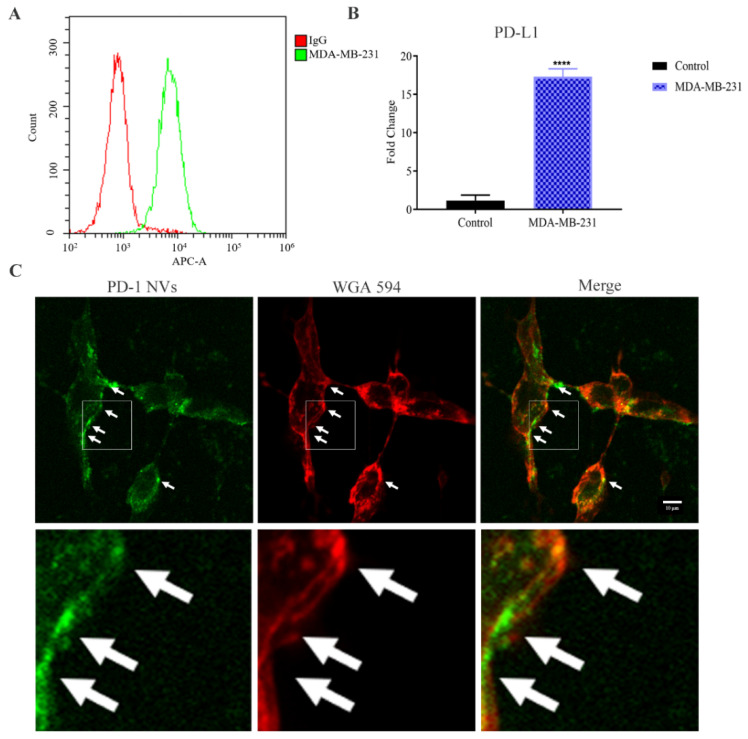
In vitro PD-1 NVs interacted with MDA-MB-231 cell. (**A**) FACS indicated the cell surface expression of PD-L1 in MDA-MB-231 cell line. (**B**) RT-qPCR assay exhibited the expression of PD-L1 in the MDA-MB-231 cell line and MCF-7 (non-TNBC cells). Data were expressed as mean ± SEM, *n* = 3. **** *p* < 0.0001. (**C**) GFP-PD-1 NVs bound with the cell membrane of MDA-MB-231 cancer cell. PD-1 NVs (50 µg/mL, protein weight) were incubated with MDA-MB-231 cancer cell for 2 h. Arrows pointed to PD-1 (on NVs), the MDA-MB-231 cell membrane (expressing PD-L1) and colocalization, respectively. WGA Alexa-Fluor 594 dye was used to detect MDA-MB-231 cell membrane (Scar bar: 10 µm).

We first assessed the expression of PD-L1 in the breast cancer cells MDA-MB-231 via FACS. PD-L1 was highly expressed in the MDA-MB-231 triple-negative breast cancer cell lines (TNBC) (Figure 4A), which was also in line with the results of previous reports. Compared to the MCF-7 as a non-TNBC cell line (control), we found that the expression of PD-L1 mRNA in MDA-MB-231 cells was 15 folds higher using RT-qPCR analysis, suggesting that the constructed PD-1&GEM NVs could target TNBC (Figure 4B) (Table 1). To test whether PD-1-GFP NVs can bind to PD-L1 on the surface of MDA-MB-231, we co-incubated the PD-1 NVs with MDA-MB-231 for 2 h and detected it by confocal microscopy. As shown in Figure 4C, PD-1-GFP NVs (green fluorescence) showed the obvious co-localization, suggesting that PD-1 NVs might competitively and specifically block the binding of PD-L1 on TNBC cell membrane to PD-1 of T cells, and restart the T cell immune killing effect.

### 3.3. Gemcitabine Inhibited the Tumor Cell Growth and PD-1&GEM NVs Induced Cell Apoptosis of Breast Cancer In Vitro

**Figure 5:**
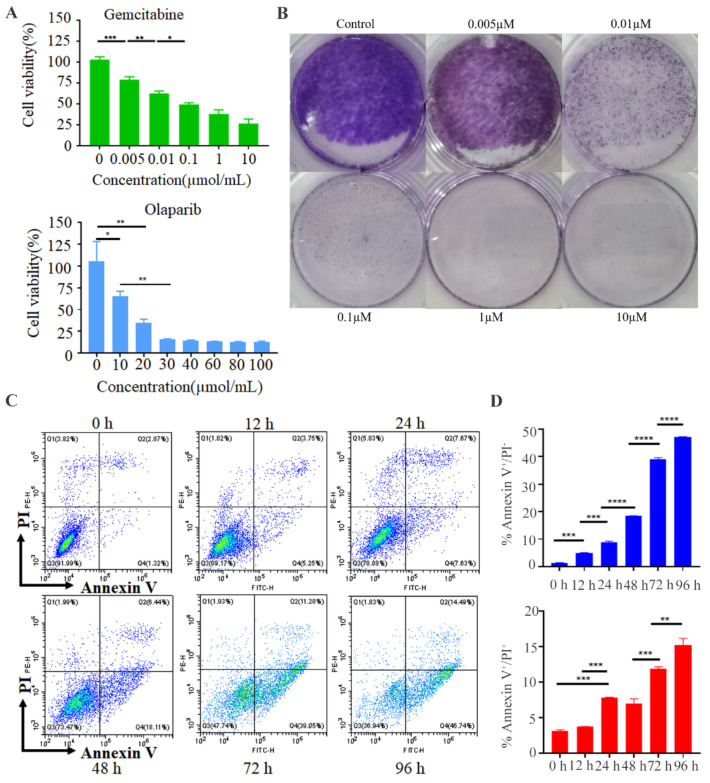
GEM inhibited the proliferation of tumor cells and PD-1&GEM NVs promoted cell apoptosis. (**A**) Cell cytotoxicity of gemcitabine and olaparib on MDA-MB-231 breast cancer cell line. (**B**) The cell clone estimated the inhibition of gemcitabine on MDA-MB-231 cell line. (**C**) FACS assay exhibited PD-1&GEM NVs induced cell apoptosis. (**D**) Corresponding statistic data measured the proportion of PI^+^/PI^−^ apoptosis cell. Data were expressed as mean ± SEM, *n* = 3. * *p* < 0.05, ** *p* < 0.01, *** *p* < 0.001, **** *p* < 0.0001.

At the beginning, the DNA-damaging chemotherapeutic drug including DNA-synthesis inhibitor gemcitabine and PARP (poly adenosine diphosphate ribose polymerase) inhibitor olaparib were both considered as a therapeutic drug. In order to verify the effect of gemcitabine and olaparib, cell activity and cell cloning experiments were performed in a dose-dependent manner. As shown in Figure 5A,B, we determined the effect of increasing concentrations of GEM (0.005 µM, 0.01 µM, 0.1 µM, 1 µM, and 10 µM) on MDA-MB-231 breast cancer cells inhibition. Results indicated that 0.1 µM, 1 µM, and 10 µM gemcitabine presented similar stronger inhibition on TNBC cells than low concentrations (0.005 µM, 0.01 µM). Importantly, the effect of 0.1 µM gemcitabine on MDA-MB-231 inhibition was comparable to the treatment with 1 µM and 10 µM, thus 0.1 µM were chosen for suitable concentration. However, cell activation experiments showed that a larger olaparib dose (30 µM) was required to achieve the single drug-killing tumors (Figure 5A). Thus, low-dose 0.1 µM gemcitabine was chosen for following experiments. Furthermore, research has revealed that gemcitabine possesses promising effects on chemotherapy in patients with breast cancer [25]. Afterwards, we developed a combined immunotherapy and chemotherapy therapy of PD-1 NVs carrying gemcitabine by the effect of targeting and carrier of NVs. Next, coated nanovesicles (PD-1&GEM NVs) were constructed by means of electroporation, and performed the experiments of GEM encapsulation and in-vitro drug release in PD-1 NVs. The result showed that gemcitabine was packaged into PD-1 nanovesicles by electroporation with an encapsulation efficiency of 21%, and the eventually released gemcitabine concentration was chosen to be 0.1 µM in vitro, (Figure S1A). We also examined the release of encapsulated GEM from PD-1&GEM NVs at different times (1 h, 4 h, 8 h, 16 h, 24 h) by ultraviolet-visible spectroscopy (UV-Vis). The results suggested that with the increase of incubation time, GEM could be gradually released from NVs with a peak time at 24 h (Figure S1B). Many studies have shown that gemcitabine can inhibit tumor cells and induce apoptosis due to its blocking of the G/S phase of DNA [26,27]. Excitedly, the early apoptosis rate (Annexin V^+^/PI^−^) of MDA-MB-231 treated with PD-1&GEM NVs was 5.25%, 7.63%, 18.11%, 39.05%, and 46.74% at 12 h, 24 h, 48 h, 72 h, and 96 h, respectively. The late apoptosis rate (Annexin V^+^/PI^+^) of MDA-MB-231 was 3.75%, 7.67%, and 11.28%, and 14.49% at 12 h, 24 h, 72 h, and 96 h, respectively, indicating that PD-1&GEM NVs possessed a significant time-dependent pro-apoptotic effect (Figure 5C,D). In conclusion, the GEM could suppress the proliferation of MDA-MB-231 and PD-1&GEM NVs could promote the apoptosis of MDA-MB-231 breast cancer cells in vitro.

### 3.4. PD-1&GEM NVs Promoted PBMC Activation and MDA-MB-231 Cell Apoptosis When Co-Cultured

**Figure 6:**
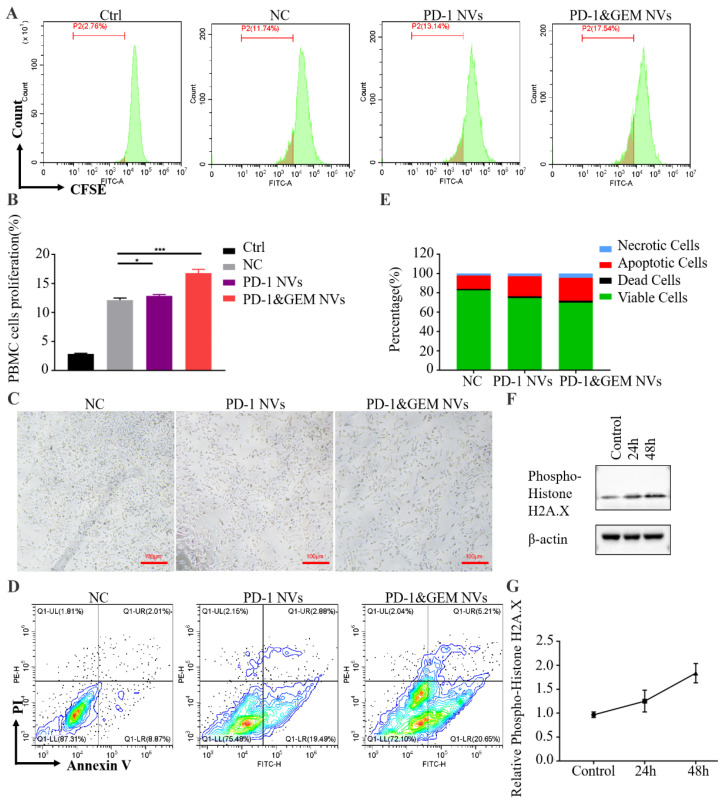
PD-1&GEM NVs promoted the apoptosis of MDA-MB-231 and activated the proliferation of PBMC cells in vitro. (**A**) Flow cytometry analysis of the proliferation of PBMC when co-cultured with MDA-MB-231 in groups received different treatments (Ctrl, NC, PD-1 NVs, and PD-1&GEM NVs) for 3 days. Control group: PBMC at day 0, NC group: free NVs. (**B**) The corresponding quantitative analysis of PBMC from different treatment groups (*n* = 3). (**C**) Microscopic examination estimated that the proliferation of MDA-MB-231 in groups received different treatments (NC, PD-1 NVs and PD-1&GEM NVs together with PBMC). (**D**) Flow cytometry analysis of the apoptosis of MDA-MB-231, which were co-cultured with PBMC. (**E**) Column data estimated the proportion of viable cell, apoptosis cell, necrotic cell, and dead cell. (**F**,**G**) Representative western blot plots and quantitative analysis of the effect of GEM at different time points on the expression of γ-H2AX, β-actin was used as a loading control. Data were expressed as mean ± SEM, *n* = 3. NS: no significant, * *p* < 0.05, *** *p* < 0.001.

In order to further study the mechanism of PD-1&GEM NVs inhibiting tumor growth and whether it affected the activity of immune cells, we extracted PBMCs from peripheral blood mononuclear cells of healthy people. PBMCs were co-incubated with human MDA-MB-231 cells to simulate the tumor immune microenvironment. As shown in Figure 6A,B, compared to the control group, the degree of PBMC proliferation was significantly promoted by 10.38%, 14.78% in the PD-1 NVs, and PD-1&GEM NVs treatment groups, suggesting that PD-1 NVs effectively relieved the negative regulation of PD-L1 expressed by tumor cells on immune cells. It was worth noting that PD-1&GEM NVs induced the most significant proliferation of PBMC cells, and maybe PBMC was activated by released antigen when combined gemcitabine killed tumor cells. Expectedly, PD-1&GEM NVs produced a better inhibitory effect on the proliferation of MDA-MB-231 cells than only PD-1 NVs treatment in the co-culture of tumor and PBMC (Figure 6C). Correspondingly, we also observed more apoptotic and dead cells in the PD-1&GEM NVs than PD-1 NVs via flow cytometry analysis (Figure 6D,E). To further explore the role of gemcitabine in cell growth inhibition and apoptosis, we examined DNA damage marker γ-H2A.X expression in MDA-MB-231 cells. Results showed that the expression of γ-H2A.X was significantly upregulated in a time-dependent manner after PD-1&GEM NVs treatment (Figure 6F,G).

Thus, these results suggest that PD-1&GEM NVs could enhance the activation of immune cells, inhibit tumor DNA synthesis, and promote tumor cell apoptosis of MDA-MB-231 cells in vitro.

### 3.5. GEM&PD-1 NVs Inhibited the Growth of TNBC Breast Cancer In Vivo

The toxicity of Free NVs and PD-1 NVs in this study was evaluated. The result showed that heart, liver, spleen, lung, and kidney showed no pathological phenomena compared to the untreated group by hematoxylin-eosin (H&E) analysis (Figure S2).

**Figure 7:**
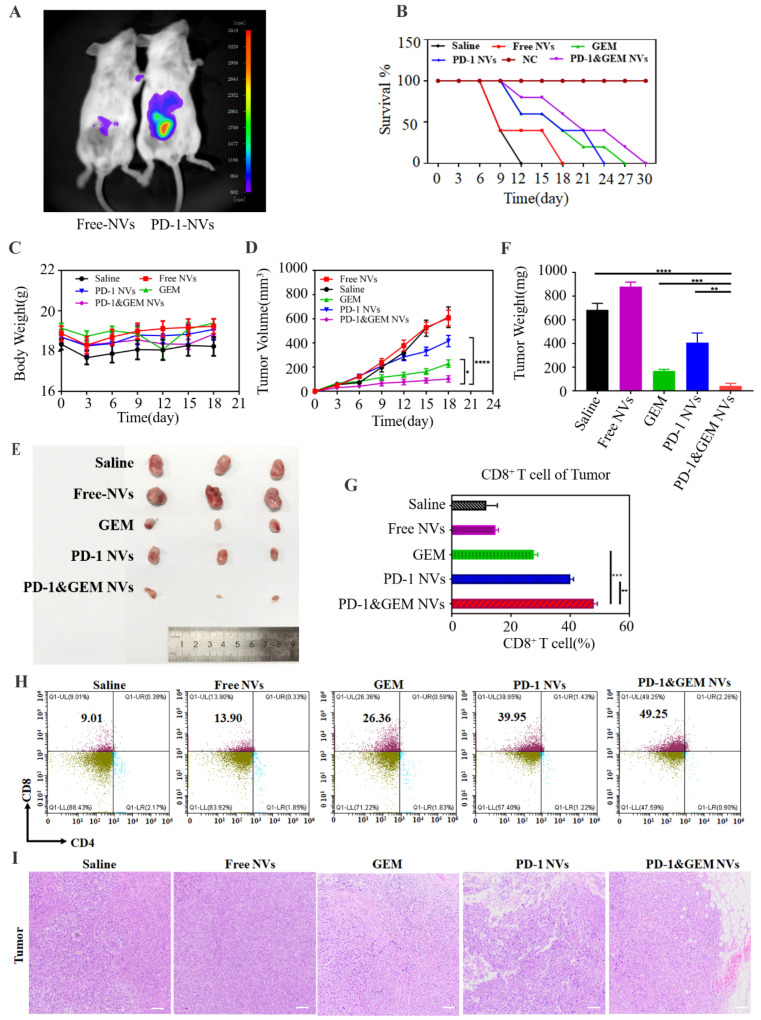
In vivo targeting ability and antitumor effect of PD-1&GEM NVs. (**A**) In vivo biodistribution imaging of PD-1 NVs that accumulate on the tumor compared to Free NVs. (**B**) Survival curves for the BALB/c mouse inoculated with TNBC received treatment of different groups (*n* = 5). Saline (Group 1), Free NVs (Group 2), GEM (Group 3), PD-1 NVs (Group 4), PD-1&GEM NVs (Group 5). (**C**) Body weight of the BALB/c mouse inoculated with TNBC received treatment of different groups (*n* = 5). Saline, Free NVs, GEM, PD-1 NVs, and PD-1&GEM NVs. (**D**) Average tumor volumes of mice inoculated with TNBC in different groups (*n* = 5). (**E**) Representational tumor image collected from euthanized mice after different treatments. Saline, Free NVs, GEM, PD-1 NVs, and PD-1&GEM NVs. (**F**) Quantitative analysis of tumor weight of different groups (*n* = 3). (**G**,**H**) Representative plots and quantitative analysis of CD8^+^ T cells (gated on positive CD3^+^ cells) in tumor in differently treated mice groups by flow cytometry (*n* = 3). Error bar, mean ± SEM. (**I**) Histological images for H&E staining obtained from the tumor of mice treated in different group. Data were expressed as mean ± SEM, *n* = 3. * *p* < 0.05, ** *p* < 0.01, *** *p* < 0.001, **** *p* < 0.0001.

Next, to explore whether the GEM&PD-1 NVs promoted the immune response in the TNBC model, mice bearing-TNBC tumors were injected intravenously with PBS, Free NVs (25 mg/kg, *n* = 5), PD-1 NVs (25 mg/kg, *n* = 5), GEM (10 mg/kg, *n* = 5), and GEM&PD-1 NVs (25 mg/kg, *n* = 5). To avoid a cross-species immune response, we collected mouse 3T3 L1 cells-derived PD-L1 NVs [28]. The biological distribution of PD-L1 NVs was performed in tumor-bearing mice. Notably, we found cy5.5 labeled PD-1 NVs intensively accumulated in the tumor site compared to Free NVs (Figure 7A). The mouse models of breast cancer injected with 4T1 cells were established successfully after 7 days. Then, mice were received different treatments for 10 continuous days (on the 18th day from tumor inoculation). Tumor samples were collected on the 18th day following the performed experiments. However, after the treatment cycle, we stopped the injection to observe the survival days (considered the 1st day) among the different treatments. It was found that the PD-1&GEM NVs group significantly prolonged the survival time of mice for 30 days among different treatments after stopping the treatment, while saline delayed 12 days (Figure 7B). Moreover, there was no obvious weight loss in mice treated with different nanovesicles groups, indicating that the NVs were safe in mice (Figure 7C). Importantly, the growth of TNBC tumor was evidently delayed in the PD-1&GEM NVs group, which had better outcome than GEM and PD-1 NVs (Figure 7D–F). Next, treatment with GEM, PD-1 NVs, and PD-1&GEM NVs increased the proportion of CD8^+^ T cell counts by 17.35%, 30.94%, and 40.24% in tumors treated with GEM, PD-1 NVs, and PD-1&GEM NVs compared to saline by flow cytometry analysis (Figure 7G,H). Moreover, hematoxylin-eosin (H&E) analysis also revealed more sparser tumor distribution in the PD-1&GEM NVs group than the saline group and the Free NVs group (Figure 7I). Therefore, these results demonstrated that PD-1&GEM NVs could inhibit TNBC tumor growth by increasing the infiltrated CD8^+^ T cells.

**Figure 8:**
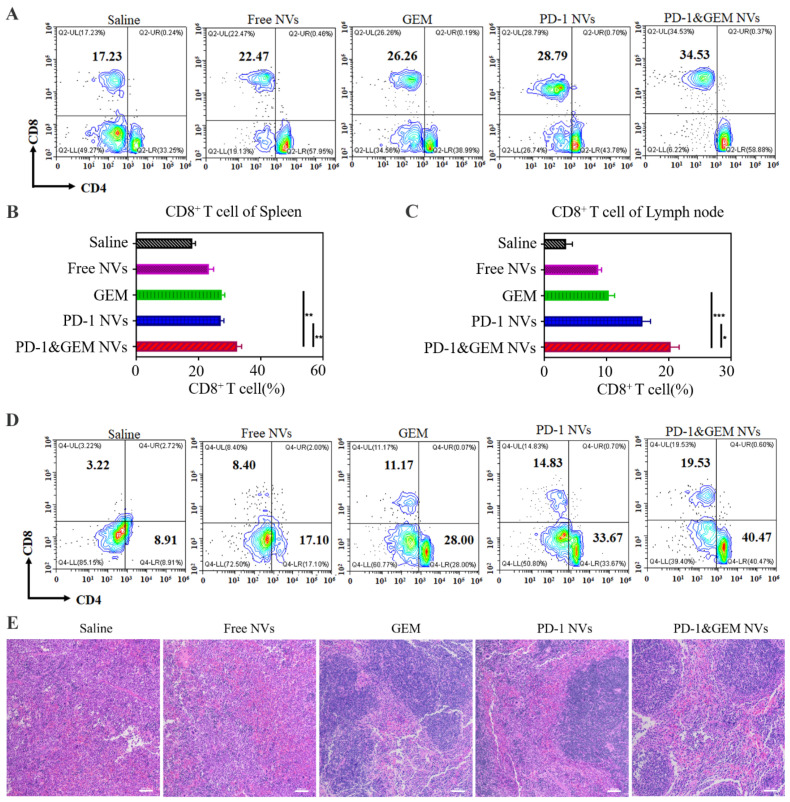
In vivo PD-1&GEM NVs promoted the density of CD8^+^ T cells in spleens and lymph nodes. (**A**,**B**) Representative plots and quantitative analysis of CD8^+^ T cells (gated on positive CD3^+^ cells) in spleens in different treated mice groups by flow cytometry (*n* = 3). Error bar, mean ± SEM. (**C**,**D**) Representative plots and quantitative analysis of CD8^+^ T cells (gated on positive CD3^+^ cells) in lymph nodes in different treated mice groups by flow cytometry (*n* = 3). Error bar, mean ± SEM. (**E**) Histological images for H&E staining obtained from the spleen of mice treated in different group. Data were expressed as mean ± SEM, *n* = 3. * *p* < 0.05, ** *p* < 0.01, *** *p* < 0.001. scale bar = 100 µm.

To further assess the role of PD-1&GEM NVs in CD8^+^ T cells, we also analyzed the activation of CD8^+^ T cells in the mice spleens. Promisingly, compared with the saline, GEM single-drug group, PD-1 NVs group and PD-1&GEM NVs group significantly prompted the proportion of CD8^+^ T cells by 9.03%, 11.56%, and 18.3%, suggesting that PD-1&GEM NVs had higher activation of CD8^+^ T cells than GEM single-drug and PD-1 NVs in the spleens (Figure 8A). This was consistent with the corresponding statistical analysis (Figure 8B). Unsurprisingly, increased CD8^+^ T cells in lymph nodes treated with GEM group, the PD-1 NVs group, and PD-1&GEM NVs group was also observed (Figure 8C,D). Similarly, hematoxylin-eosin (H&E) analysis also revealed that more normal cell morphologies were detected in spleens collected from mice received with the GEM group, the PD-1 NVs group, and the PD-1&GEM NVs treatments, compared with the pathological state in the normal saline group and the Free NVs group (Figure 8E). These results indicated that PD-1&GEM NVs restrained the proliferation of breast cancer and promoted CD8^+^ T cell activation in vivo.

## 4. Discussion

TNBC is considered as an aggressive type of breast cancer because it grows and spreads quickly and lacks effective treatment options, thereby leading to a poor prognosis [29]. Recently, research has found that combination therapies displayed favorable efficacy of treatment [30]. Excitingly, combination with chemotherapy drugs and immunotherapies can improve clinical outcomes in patients [31,32]. In 14 November 2020, the FDA approved PD-1 inhibitor Keytruda combined with chemotherapy for TNBC. This is the first time that combination therapy of immunotherapy and chemotherapy has been approved by FDA for the treatment of breast cancer, representing an important milepost [13]. However, another similar treatment scheme using PD-L1 inhibitor Tecentriq combined with albumin paclitaxel was rejected by EMA because it did not get the expected effect in the phase III clinical trial in January 2021 [33]. It is still very necessary to explore new combination therapies of different immune drugs and chemotherapeutic drugs. Therefore, exploring different combination therapies still has potential significance for the treatment of the triple-negative breast cancers.

Synergistic nanovesicles as a drug carrier could deliver anti-tumor drugs in a targeted manner, such as liposomes. Although liposomes have been applied in clinic trials by better compatibility and modifications, poor targeting, unstable morphology, immunogenicity, and their inability to cross the biological barrier have limited their therapeutic efficacy [34]. Recently, exosomes naturally released by different cells as drug carrier have become an alternative strategy by overcoming the abovementioned shortcomings of liposomes. However, complex purification processes and limited mass production are the obstacles of clinical translation. Strikingly, membrane-derived nanovesicles (NVs) share similar characteristics and function with exosomes, which have advantages in large-scale preparation, and can at the same time stably carry targeting ligands through genetic modification strategies and carry clinically chemotherapeutic drugs [21,22]. Noteworthily, it has been reported that NVs could retain structural stability for over 7 days at 4 °C as the storage condition, and the zeta and diameter of NVs still retain stability for over 24 h when in physiological conditions (PBS +10% serum) [35]. In this study, we proposed a protocol for cell-membrane derived PD-1 NVs as a GEM-targeted drug delivery system. PD-1 NVs could not only passively accumulate in the tumor due to the enhanced permeability and retention (EPR) effect [36,37], but also actively target overexpressing-PD-L1 TNBC, contributing to enrichment of PD-1 NVs. We also confirmed that the PD-1 NVs could efficiently bind to the PD-L1 receptor on breast cancer cells in vitro (Figure 4C). In addition, in vivo experiments also observed that the cell vesicles with high expression of PD-1 were more enriched in the transplanted tumor site of MDA-MB-231 than PD-1 free vesicles (Figure 7A). As we know that most chemotherapeutic drugs have low selectivity, long-term high-dose use will cause a great toxic effect and other side effects. Thus, wrapped gemcitabine in PD-1 NVs will improve the pharmacokinetics, increase the effective drug concentration at the tumor site, and significantly reduce the systemic toxicity of gemcitabine. Recent studies have emphasized that when cell-membrane derived NVs fused with cell membranes by means of lipid fusion, encapsulated contents in the NVs were delivered directly to the cytoplasm. In addition, NVs were also internalized by phagocytosis-based uptake pathway. After cellular uptake, NVs may be transported to endosomes and subsequently fused with lysosomes for degradation of NVs. Meanwhile, packaged drugs from NVs were gradually released into the cytoplasm, thus leading to enhanced drug delivery with better safety profiles [21,38]. Moreover, if gemcitabine within the NVs was released outside the tumor cells and subsequently penetrated into the tumor cytoplasm, they thereby improved the therapeutic target effect of the encapsulated drug at the tumor sites. It was noteworthy that our results demonstrated that gemcitabine from PD-1&GEM NVs released slowly with a peak of 24 h in vitro (Figure S1B). In brief, the combined NVs strategy outperformed the efficacy of gemcitabine when administered alone.

Of note, our study also verified that PD-1 NVs combined with gemcitabine has better therapeutic effect. PD-1&GEM NVs showed a better inhibition on the proliferation of TNBC tumor than PD-1 NVs alone, achieving a synergetic effect of immunotherapy and chemotherapy. Moreover, PD-1&GEM NVs significantly promoted the proliferation of PBMC, and perhaps PBMC was activated by released antigen when gemcitabine killed the tumor cells. For example, the proliferation of CFSE-staining PBMC treated with PD-1&GEM NVs was most significant among different treatment groups (Figure 6A). Consistent with in vitro experiments, in vivo PD-1&GEM NVs remarkably increased infiltrated CD8^+^ T cell and delayed the tumor growth more than PD-1 NVs (Figure 7D,H). Therefore, our study highlighted the power of combining low-dose gemcitabine and PD-1 in the nanovesicles as treatment to inhibit the proliferation of triple-negative breast cancer.

## Figures and Tables

**Table 1:** qPCR primers sequences.

Genes	Forward Primer Sequence 5′→3′	Reverse Primer Sequence 5′→3′
Mouse-*β-Actin* Mouse-*Pd-l1* Human-*Pd-1* Human-*Pd-l1*	GGCTGTATTCCCCTCCATCG TCTGATCGTCGATTGGCAGC CCCAAGGCGCAGATCAA GGTGAGGATGGTTCTACACAG	CCAGTTGGTAACAATGCCATGT CGTTGTTCCAGGCTCCTCTC CTGGCGAGCCTTAGTTTGGAC GAGAACTGCATGAGGTTGC
Human-*β-Actin*	CCACACTGTGCCCATCTAC	AGGATCTTCATGAGGTAGTCAGTC

## Data Availability

All data generated or analyzed during this study are included in this published article and its Supplementary Information Files.

## Supplementary Materials

The following supporting information can be downloaded at: https://www.mdpi.com/article/10.3390/pharmaceutics14061263/s1.
