# Supplementary material for: PD-1 Cellular Nanovesicles Carrying Gemcitabine to Inhibit the Proliferation of Triple Negative Breast Cancer Cell
Source: Pharmaceutics. 2022 Jun 14;14(6):1263. doi: 10.3390/pharmaceutics14061263 (PMC9229990; doi:10.3390/pharmaceutics14061263)
Supplement: Supplementary file 1 [file pharmaceutics-14-01263-s001.zip › pharmaceutics-1706837-supplementary.pdf]

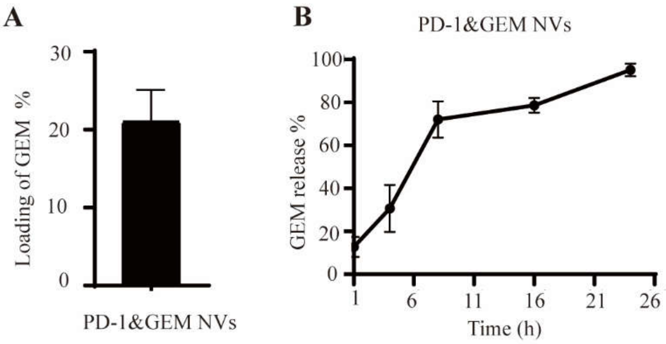

Figure S1. In vitro loading of release of GEM-carried PD-1 NVs. (**A**) The capacity of GEM-loaded PD-1 NVs by electroporation (n = 3). (**B**) The release profiles of GEM from PD-1 NVs at different time (1 h, 4 h, 8 h, 16 h, 24 h) (n = 3).

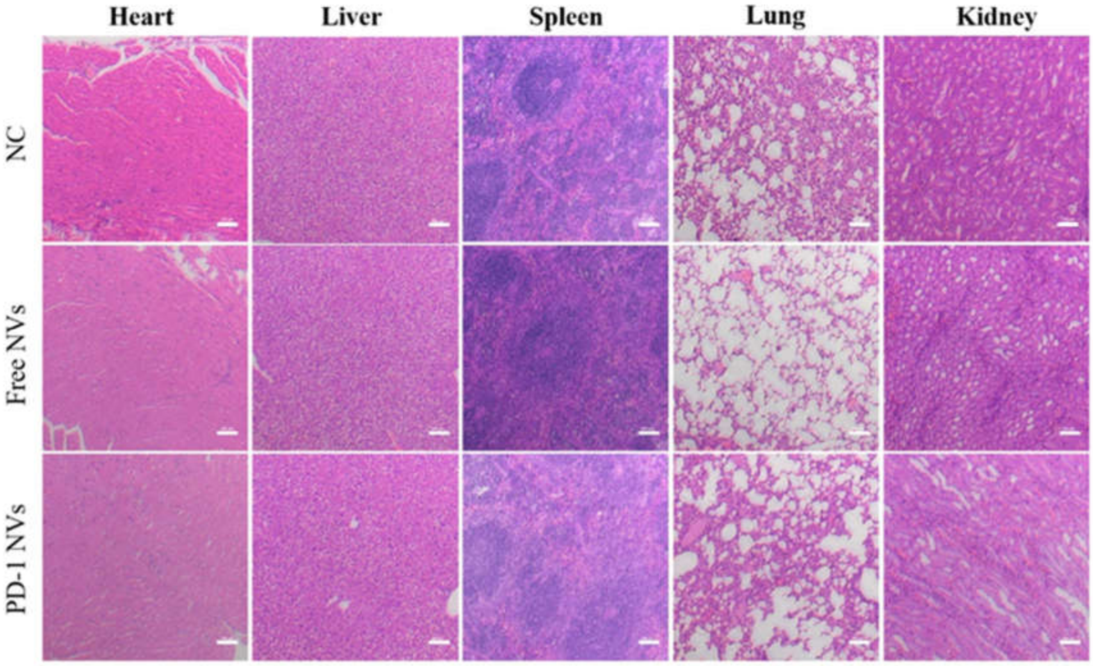

Figure S2. In vivo toxicity tests for PD-1 NVs. Histological images for H&E staining obtained from the lung, heart, kidney, spleen and liver of mice treated with different NVs at day 20 post-injection. Scale bar: 100 μm.
